# Impact of chronic fibrosing interstitial lung disease on healthcare use: association between fvc decline and inpatient hospitalization

**DOI:** 10.1186/s12890-023-02637-8

**Published:** 2023-09-09

**Authors:** David Singer, Benjamin Chastek, Andrew Sargent, Jonathan C. Johnson, Sharash Shetty, Craig Conoscenti, Elana J. Bernstein

**Affiliations:** 1 Boehringer Ingelheim Pharmaceuticals, Inc., Ridgefield, CT USA; 2 Optum, Health Economics and Outcomes Research, Eden Prairie, MN USA; 3 Columbia University Irving Medical Center, New York, NY USA

**Keywords:** Chronic fibrosing interstitial lung disease, Healthcare resource utilization, Forced vital capacity, Lung function

## Abstract

**Background:**

Many types of interstitial lung diseases (ILDs) may transition to progressive chronic-fibrosing ILDs with rapid lung function decline and a negative survival prognosis. In real-world clinical settings, forced vital capacity (FVC) measures demonstrating progressive decline may be linked to negative outcomes, including increased risks of costly healthcare resource utilization (HRU). Thus, we assessed the relationship between rate of decline in lung function and an increase in HRU, specifically inpatient hospitalization, among patients with chronic fibrosing ILD.

**Methods:**

This study utilized electronic health records from 01-Oct-2015 to 31-Oct-2019. Eligible patients (≥ 18 years old) had ≥ 2 fibrosing ILD diagnosis codes, clinical activity for ≥ 15 months, and ≥ 2 FVC tests occurring 6 months apart. Patients with missing demographic data, IPF, or use of nintedanib or pirfenidone were excluded. Two groups were defined by relative change in percent of predicted FVC (FVC% pred) from baseline to 6 months: significant decline (≥ 10%) vs. marginal decline/stable FVC (decrease < 10% or increase). The primary outcome was defined as the occurrence of an inpatient hospitalization 6 months after the first FVC value. Descriptive and multivariable analysis was conducted to examine the impact of FVC decline on occurrence of inpatient hospitalization.

**Results:**

The sample included 566 patients: 13% (n = 75) with significant decline and 87% (n = 491) with marginal decline/stable FVC; their mean age (SD) was 65 (13.7) years and 56% were female. Autoimmune diagnoses were observed among 40% of patients with significant decline, and 27% with marginal decline/stable FVC. The significant decline group had better lung function at baseline than the marginal/stable group. For patients with FVC% <80% at baseline, reduction of FVC% ≥10% was associated with significantly increased odds of an inpatient hospitalization (odds ratio [OR] 2.85; confidence interval [CI] 1.17, 6.94 [p = 0.021]).

**Conclusion:**

Decline in FVC% ≥10% was associated with increased odds of inpatient hospitalization among patients with reduced lung function at baseline. These findings support the importance of preserving lung function among patients with fibrosing ILD.

**Supplementary Information:**

The online version contains supplementary material available at 10.1186/s12890-023-02637-8.

## Background

Interstitial lung diseases (ILDs) are a heterogeneous group of disorders characterized by inflammation and/or fibrosis of the lung parenchyma; the most commonly diagnosed form is idiopathic pulmonary fibrosis (IPF) [1, 2]. While IPF is always progressive and fibrosing, other ILDs may at some point transition from acute or inflammatory behavior to a chronic fibrosing ILD with a “progressive phenotype,” associated with rapid functional decline and increased mortality [3, 4].

Chronic fibrosing ILD may be described as a fibro-proliferative or inflammatory condition and may be associated with several autoimmune disorders, infections, or environmental exposures [5]. Furthermore, researchers have suggested that progressive non-IPF ILDs share some pathogenic mechanisms and clinical disease behavior with IPF [6], but they are generally distinguished from non-progressive ILD by continuing functional decline associated with mortality [7, 8]. Declines in forced vital capacity (FVC), a primary clinical measure of lung function, were among characteristics used to identify progression in a clinical trial setting [9].

Traditionally, many chronic fibrosing ILDs were treated with corticosteroids and immunomodulators, with only modest success [10], whereas two antifibrotic therapies were approved for treatment of IPF: nintedanib [11] and pirfenidone [12]. A Phase III trial of nintedanib (INBUILD) was conducted specifically among patients who had non-IPF chronic fibrosing ILD with a progressive phenotype [9, 13]. This study showed that the annual rate of decline in FVC was significantly lower among patients who received nintedanib, as compared with those who received placebo. In March 2020, nintedanib was approved for the treatment of chronic fibrosing ILD with a progressive phenotype.

It is important to understand the impact of declining lung function among patients with ILD in real-world clinical settings. An association has been established between FVC decline and healthcare resource utilization (HRU) outcomes in IPF [14–16], but this relationship has not been previously established for non-IPF chronic fibrosing ILD. The primary aim of this study was to assess the relationship between FVC decline and HRU—specifically, inpatient hospitalization—among patients with chronic fibrosing ILD.

## Methods

### Design and sample selection

**Fig. 1:**
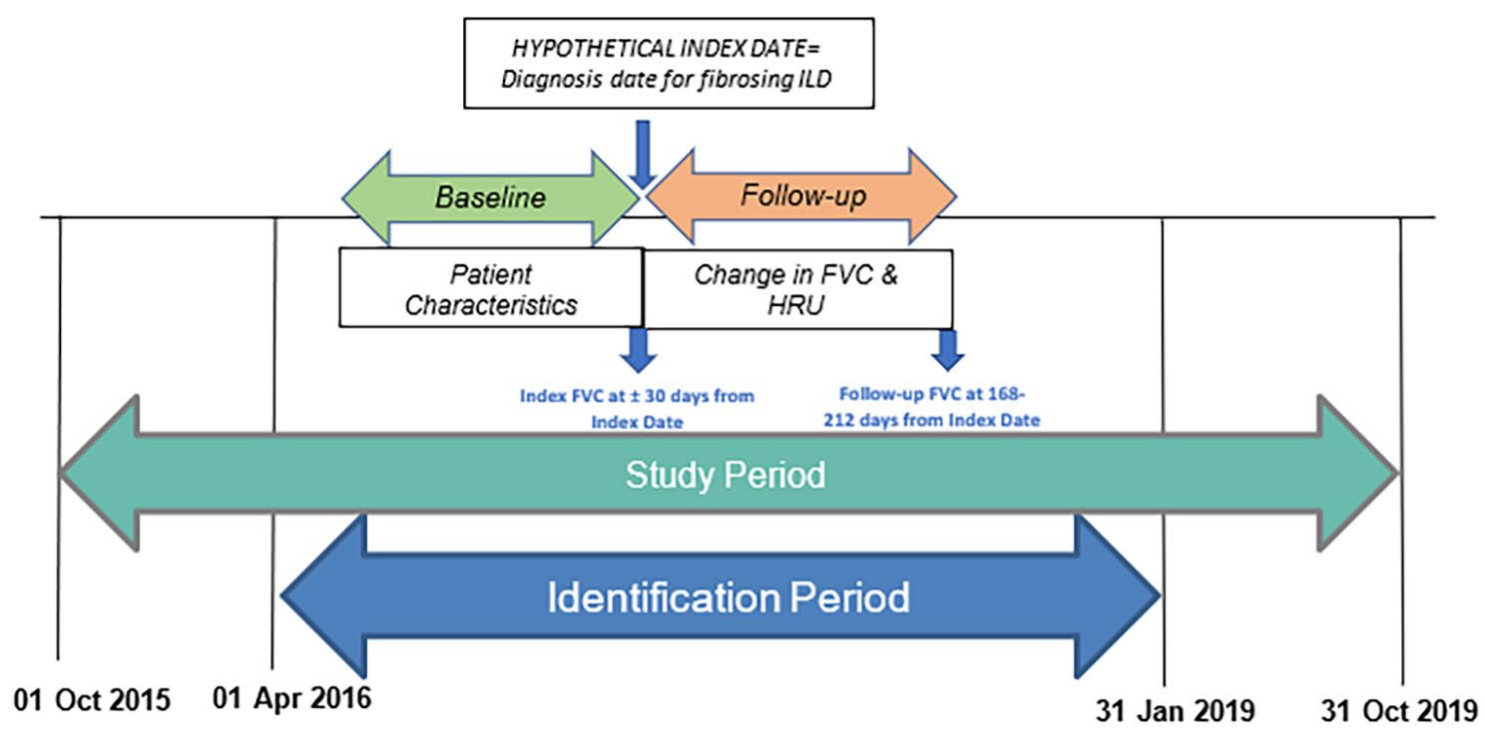
Study Design. *Note*: FVC = forced vital capacity; HRU = healthcare resource utilization; ILD = interstitial lung disease. Baseline period = time from index FVC value to 6 months prior to index FVC value; Follow-up period = time from index date until follow-up FVC value; Study period = includes the identification period plus a baseline period, and a follow-up period; Identification period = time during which index dates (date of first diagnosis) in the study period are set. The baseline period was used to describe patient demographic and clinical characteristics, comorbid conditions, and baseline HRU. The follow-up period was used to describe change in FVC and all-cause HRU

This study used electronic health records (EHR) data for the study period of 01 October 2015 through 31 October 2019 (Fig. 1). Adult patients (≥ 18 years of age) were identified by at least 2 encounters with diagnoses of fibrosing ILD (Appendix Table A1 for International Classification of Diseases Clinical Modification [ICD]-10-CM codes) during the *identification period* of 01 April 2016 to 31 January 2019. The date of the earliest ILD diagnosis code was set as the *index date*. All patients were required to have clinical activity for at least 6 months before and 9 months after the index date. Patients with at least 1 FVC test result within ± 1 month of the index date (*index FVC*), and another test result from 168 to 212 days after the index date (*follow-up FVC*) were included [procedure codes in Appendix A2]. Ranges of dates were used to maximize sample size, based upon distribution of available FVC measures for eligible patients. For patients who had multiple valid FVC measures during either the index date or follow-up, the value closest to the index date or to the follow-up FVC period was selected.

We performed a retrospective observational study using EHR data from the Optum Clinical Database (OCD). By 2018, the OCD contained data on approximately 85 million unique patients across the United States and Puerto Rico, with an average of 40 months of observed data per patient. The OCD data include details of physician office visits and hospital stays, including laboratory results, prescriptions written, medications administered in the hospital, procedures, and diagnoses, as well as physician, pathology, and radiology notes. This study utilized spirometry data obtained from structured fields and through natural language processing of provider notes.

Patients were excluded if there was any missing demographic data as of the index date; if they had a diagnosis code for IPF (ICD-10-CM J84.112) during the study period; or if there were any pharmacy orders for nintedanib or pirfenidone during the study period.

### Study measures

Baseline patient demographic and clinical characteristics were obtained during the period of 6-months pre-index through index date (date of first diagnosis). Demographic characteristics included age, sex, race, region, and insurance type. In addition, general comorbid condition categories were identified using the Clinical Classifications Software managed by the Agency for Healthcare Research and Quality (AHRQ; CCS for ICD-9-CM/ICD-10-CM [17]). Relevant underlying conditions were identified by diagnosis codes, and a Charlson Comorbidity Index score was calculated [18].

Predicted FVC (FVC_Pred_) was computed using the NHANES III definition [19], adjusted according to race/ethnicity [20, 21]. Percent of predicted FVC (FVC%) was computed for the index and follow-up FVC measures, as: (FVC_Observed_/FVC_Predicted_)*100%. Baseline and follow-up ILD-related and all-cause HRU were measured for ambulatory visits, emergency department (ED) visits, and inpatient admissions. The follow-up period included time from index FVC value until the follow-up FVC value.

### Statistical analyses

Analyses were performed based on patients’ change in FVC% status from index FVC to the 6-month FVC. Study patients were classified into two groups based on the relative severity of their change in FVC%: significant decline (decrease of ≥ 10%), and marginal decline/stable FVC (decrease < 10% or increase in FVC). A change in FVC% of at least 10% was chosen to define significant lung function decline to align with the criteria used to define progressive phenotype in the INBUILD clinical trial [9, 13].

All baseline and follow-up measures were analyzed descriptively. Count of patients and percentages described dichotomous and polychotomous variables and means, medians, and standard deviations (SD) described continuous variables. All measures were reported for the overall sample and for each group: significant decline vs. marginal decline/stable. To compare the two groups, F-test/ANOVA was used for continuous variables and Pearson chi-square test was used for binary variables.

Logistic regression was performed to examine the association between change in lung function (stratified by index FVC value < 80% versus ≥ 80%) [22] and all-cause inpatient hospitalizations, while controlling for potential confounders including age, index year, region, income, AHRQ comorbidities, concomitant medications, and baseline HRU (see full list of covariates in Appendix Table A3). A full model was developed including all available relevant measures as covariates, and a stepwise selection model was also conducted as a sensitivity analysis (Appendix Table A4). Variables were removed from the stepwise selection model if their p-value was > 0.2. All analyses were performed using SAS V9.4 (Cary, NC).

This study used data extracts that were fully de-identified and HIPAA-compliant. Thus, Institutional Review Board review and approval were not required.

## Results

### Sample description

**Fig. 2:**
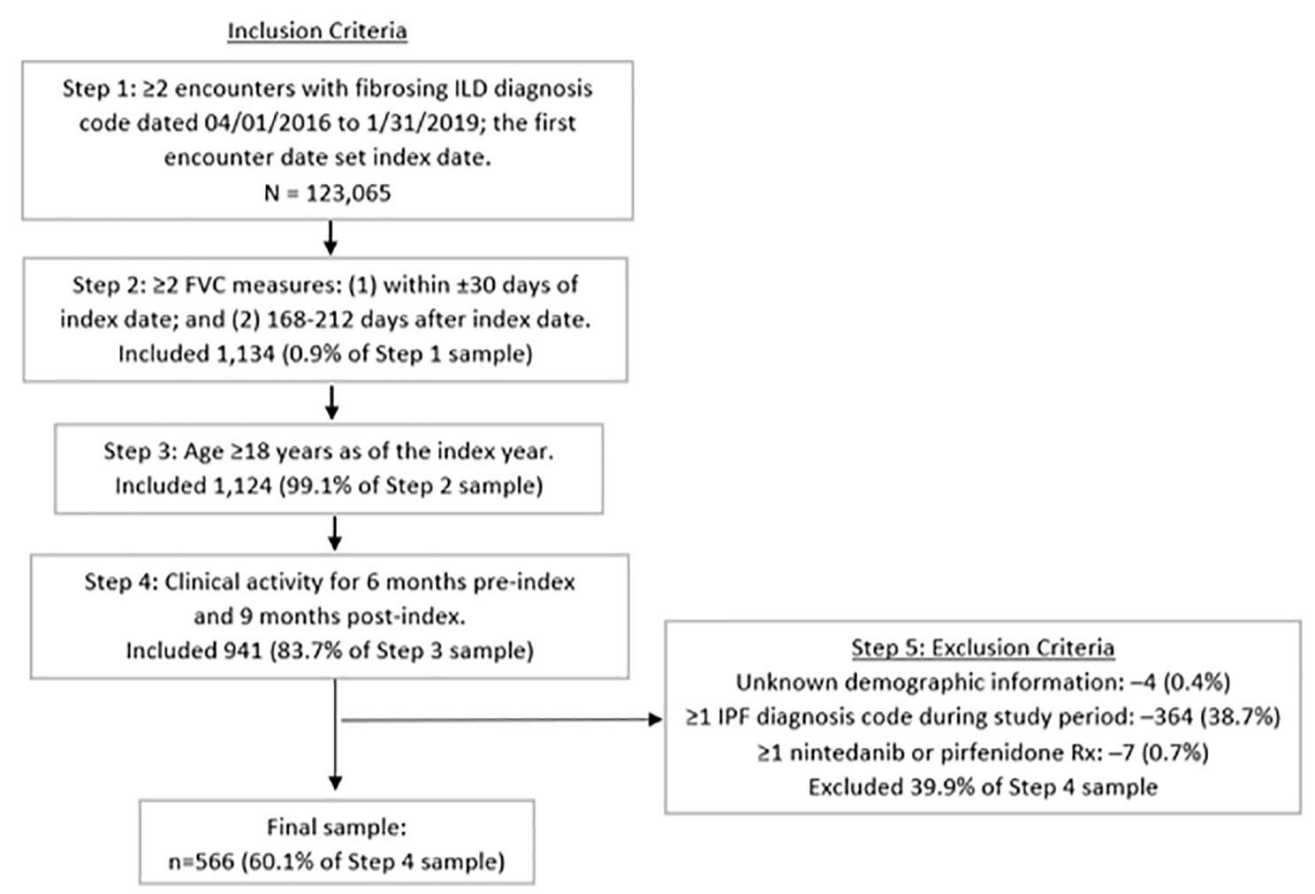
Sample Selection and Attrition.*Note*: A large drop in N was observed here because only 1,134 had FVC values in both time periods required for comparison across 6 months. FVC = forced vital capacity; ILD = interstitial lung disease, Rx = prescription

Among 123,065 patients with diagnoses for fibrosing ILD, 1,134 had 2 FVC values available in the required time period (Fig. 2). The final study population included 566 patients (Fig. 2): 75 (13%) with significant decline in FVC and 491 (87%) with marginal decline/stable FVC.

The mean (SD) age was 64.7 (13.7) years, with no significant difference between the two groups (Table 1). The majority of the study population was female (56%), non-Hispanic (92%), Caucasian (83%) and insured by commercial (30%) or Medicare (37%) plans, with no significant differences in baseline characteristics between the two groups.

**Table 1:** Demographic Characteristics by FVC Decline Groups

Demographics	Total (N = 566)	Significant Decline (N = 75)	Marginal Decline/Stable (N = 491)	p-value
Age, mean (SD)	64.7 (13.7)	66.1 (13.1)	64.5 (13.8)	0.338
Median	67.00	68.00	66.00	
Age group, n (%)				
18–29	14 (2.5)	1 (1.3)	13 (2.7)	0.495
30–39	21 (3.7)	3 (4.0)	18 (3.7)	0.887
40–49	40 (7.1)	3 (4.0)	37 (7.5)	0.266
50–59	100 (17.7)	15 (20.0)	85 (17.3)	0.570
60–69	160 (28.3)	20 (26.7)	140 (28.5)	0.741
70–79	156 (27.6)	21 (28.0)	135 (27.5)	0.927
≥ 80	75 (13.3)	12 (16.0)	63 (12.8)	0.451
Gender, n (%)				
Female	318 (56.2)	41 (54.7)	277 (56.4)	0.776
Male	248 (43.8)	34 (45.3)	214 (43.6)	0.776
Year of index date, n (%)				
2016	342 (60.4)	51 (68.0)	291 (59.3)	0.150
2017	122 (21.6)	13 (17.3)	109 (22.2)	0.340
2018	102 (18.0)	11 (14.7)	91 (18.5)	0.417
Race, n (%)				
Caucasian	469 (82.9)	65 (86.7)	404 (82.3)	0.348
African American	69 (12.2)	9 (12.0)	60 (12.2)	0.957
Asian	4 (0.7)	0 (0.0)	4 (0.8)	0.433
Other/Unknown	24 (4.2)	1 (1.3)	23 (4.7)	0.180
Ethnicity, n (%)				
Hispanic	15 (2.7)	1 (1.3)	14 (2.9)	0.466
Non-Hispanic	523 (92.4)	73 (97.3)	450 (91.7)	0.084
Other/Unknown	28 (5.0)	1 (1.3)	27 (5.5)	0.121
US Census Region, n (%)				
Northeast	159 (28.1)	14 (18.7)	145 (29.5)	0.051
Midwest	317 (56.0)	50 (66.7)	267 (54.4)	0.046
South	48 (8.5)	8 (10.7)	40 (8.2)	0.466
West	29 (5.1)	3 (4.0)	26 (5.3)	0.636
Other/Unknown	13 (2.3)	0 (0.0)	13 (2.7)	0.154
Insurance Type, n (%)				
Commercial	169 (29.9)	21 (28.0)	148 (30.1)	0.706
Medicaid	23 (4.1)	1 (1.3)	22 (4.5)	0.199
Medicare	208 (36.8)	32 (42.7)	176 (35.9)	0.254
Commercial/Medicaid	14 (2.5)	3 (4.0)	11 (2.2)	0.361
Commercial/Medicare	104 (18.4)	12 (16.0)	92 (18.7)	0.569
Medicare/Medicaid	15 (2.7)	1 (1.3)	14 (2.9)	0.446
Commercial/Medicare/Medicaid	5 (0.9)	0 (0.0)	5 (1.0)	0.380
Uninsured	6 (1.1)	1 (1.3)	5 (1.0)	0.804
Missing/Unknown	22 (3.9)	4 (5.3)	18 (3.7)	0.487

*Note*. FVC = forced vital capacity; SD = standard deviation; US = United States

### Clinical characteristics

The mean Charlson comorbidity score of the study population was 1.62 (± 1.71), with no statistically significant differences between the two groups (Table 2). The most common types of autoimmune ILDs were rheumatoid arthritis (8.5%) and systemic sclerosis (5.0%). Common comorbid conditions of interest prevalent in the study population included chronic obstructive pulmonary disease (33.6%), obstructive sleep apnea (18.7%), asthma (15.0%), heart failure (13.1%), and pulmonary hypertension (12.2%). No statistically significant differences were observed between groups in comorbid conditions. Baseline ED encounters occurred among 16.6% and inpatient hospitalizations among 23% of patients. There were no differences in baseline HRU between the two groups. The most common baseline medications included corticosteroids (38.9%) and histamine H2 receptor antagonists/proton pump inhibitors (29.0%).

The mean (SD) index FVC% for the overall study population was 80.2 (28.0). The significant decline group had higher mean (SD) index FVC% [88.5 (31.4)] than the marginal decline/stable FVC group [78.9 (27.3)]; p = 0.006. The proportion of patients with FVC% ≥80% was higher in the significant decline group than in the marginal decline/stable FVC group (61.3% vs. 40.7%, p < 0.001). The proportion of patients with FVC% of 70% to < 80% was lower in the significant decline group than in the marginal decline/stable FVC group (9.3% vs. 18.9%, p = 0.042).

**Table 2:** Clinical Characteristics by FVC Decline Groups

	Total (N = 566)	Significant Decline (N = 75)	Marginal Decline/Stable (N = 491)	p-value
Baseline Charlson comorbidity score, mean (SD)	1.62 (1.71)	1.85 (1.66)	1.59 (1.72)	0.209
Autoimmune ILDs, n (%)				
Rheumatoid arthritis	48 (8.5)	5 (6.7)	43 (8.8)	0.545
Sarcoidosis	40 (7.1)	9 (12.0)	31 (6.3)	0.073
Systemic sclerosis	28 (5.0)	5 (6.7)	23 (4.7)	0.461
Lupus	18 (3.2)	5 (6.7)	13 (2.7)	0.065
Mixed connective disease	14 (2.5)	1 (1.3)	13 (2.7)	0.495
Sjögren’s syndrome	9 (1.6)	2 (2.7)	7 (1.4)	0.424
Dermatomyositis/polymyositis	7 (1.2)	3 (4.0)	4 (0.8)	0.020
Comorbid Conditions, n (%)				
COPD	190 (33.6)	27 (36.0)	163 (33.2)	0.632
Obstructive sleep apnea	106 (18.7)	17 (22.7)	89 (18.1)	0.348
Asthma	85 (15.0)	11 (14.7)	74 (15.1)	0.927
Heart failure	74 (13.1)	11 (14.7)	63 (12.8)	0.660
Pulmonary hypertension	69 (12.2)	7 (9.3)	62 (12.6)	0.417
Lung cancer	11 (1.9)	2 (2.7)	9 (1.8)	0.626
Baseline HRU measures, n (%)				
All-cause ambulatory visit, 6 months pre-index	509 (89.9)	69 (92.0)	440 (89.6)	0.522
All-cause ED visit, 6 months pre-index	94 (16.6)	14 (18.7)	80 (16.3)	0.607
All-cause IP stay, 6 months pre-index	130 (23.0)	16 (21.3)	114 (23.2)	0.718
All-cause IP stay (with ICU), 6-months pre-index*	20 (15.4)	3 (18.8)	17 (14.9)	0.690
Baseline medications, n (%)				
Corticosteroid	220 (38.9)	32 (42.7)	188 (38.3)	0.469
H_2_-antagonists and PPIs	164 (29.0)	18 (24.0)	146 (29.7)	0.308
Mycophenolate mofetil	34 (6.0)	5 (6.7)	29 (5.9)	0.796
Tacrolimus	27 (4.8)	2 (2.7)	25 (5.1)	0.359
ERAs, PDE-5s, prostacyclins, sGCs	13 (2.3)	1 (1.3)	12 (2.4)	0.550
Azathioprine	8 (1.4)	3 (4.0)	5 (1.0)	0.042
Rituximab	6 (1.1)	0 (0.0)	6 (1.2)	0.336
Cyclophosphamide	3 (0.5)	0 (0.0)	3 (0.6)	0.497
Cyclosporine	0 (0.0)	0 (0.0)	0 (0.0)	-
Index FVC percent predicted, mean (SD)	80.2 (28.0)	88.5 (31.4)	78.9 (27.3)	0.006
FVC percent predicted, categorical, n (%)				
≥ 80%	246 (43.5)	46 (61.3)	200 (40.7)	< 0.001
70- <80%	100 (17.7)	7 (9.3)	93 (18.9)	0.042
< 70%	220 (38.9)	22 (29.3)	198 (40.3)	0.069
BMI, n	383	48	335	
Mean (SD)	33.6 (6.9)	34.0 (6.8)	33.6 (6.9)	0.675

*Note*. BMI = body mass index; COPD = chronic obstructive pulmonary disease; ED = emergency department; ERA = endothelin receptor antagonists; FVC = forced vital capacity; ILD = interstitial lung disease; IP = inpatient; PDE = phosphodiesterase; PH = pulmonary hypertension; PPI = proton pump inhibitors; SD = standard deviation; sGC = guanylate cyclase stimulators. *among those with an IP stay

### Unadjusted analyses of HRU outcomes

Among the significant decline group, 37% of patients had an inpatient hospitalization, as compared with 30% of the marginal decline/stable FVC group (p = 0.21). In addition, no statistically significant differences were observed in ambulatory or ED visits between the two groups (Table 3).

**Table 3:** HRU Outcomes by FVC Decline Groups

	Total (N = 566)	Significant Decline (N = 75)	Marginal Decline/ Stable FVC (N = 491)	p-value
All-cause ambulatory visit, n (%) All-cause ambulatory visit count, mean (SD)	564 (99.6) 4.9 (4.8)	75 (100.0) 4.7 (4.8)	489 (99.6) 5.0 (4.8)	0.580 0.619
All-cause ED visit, n (%)	95 (16.8)	14 (18.7)	81 (16.5)	0.640
All-cause IP hospitalization, n (%)	176 (31.1)	28 (37.3)	148 (30.1)	0.210

*Note.* ED = emergency department; FVC = forced vital capacity; HRU = healthcare resource utilization; IP = inpatient

### Multivariable analyses of inpatient hospitalization

**Fig. 3:**
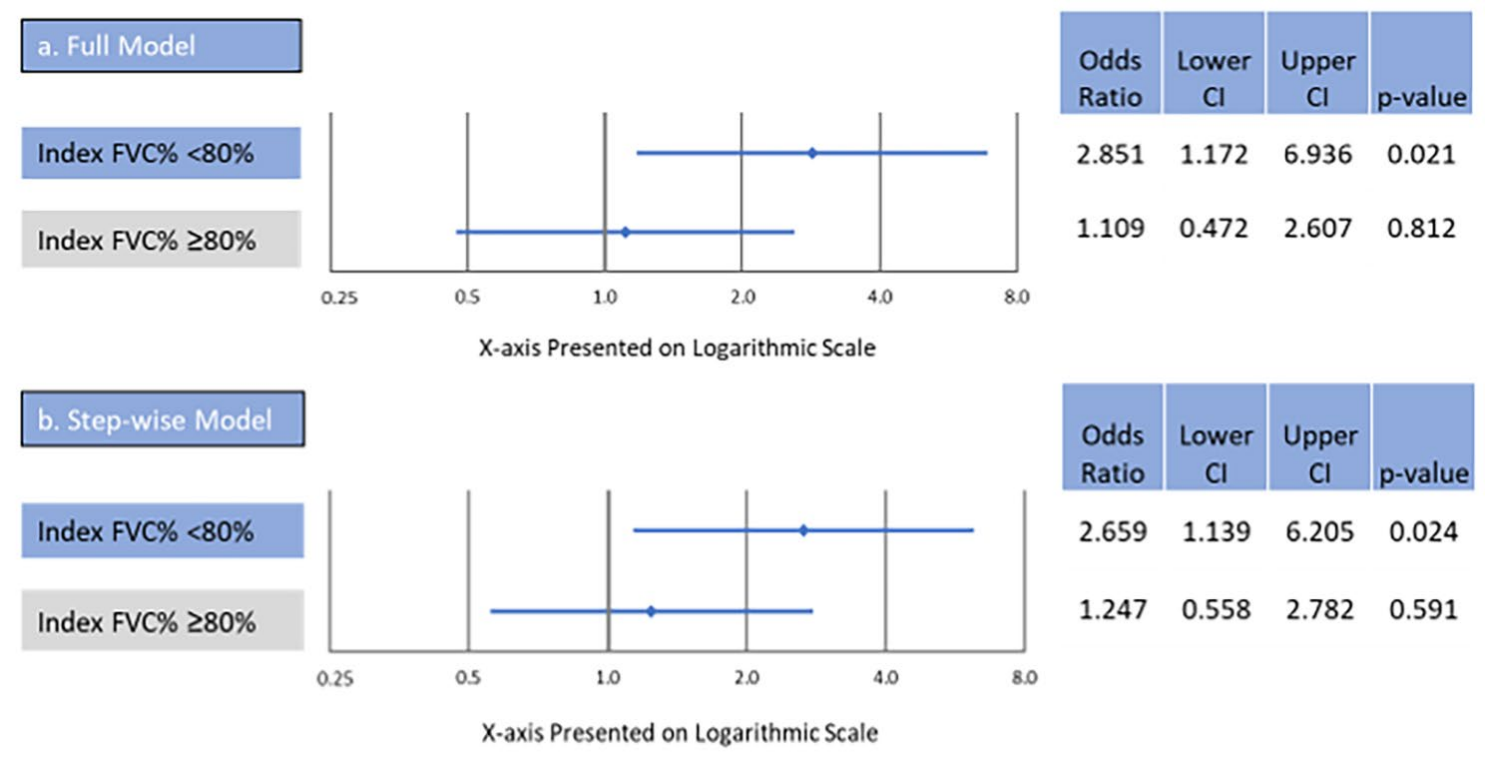
Logistic Regression (a and b) of All-Cause Inpatient Hospitalization. *Note*: Values to the right of 1 indicate higher odds of inpatient hospitalization for patients with significant decline compared to patients with marginal decline/stable FVC. CI = 95% confidence interval; FVC = forced vital capacity

Logistic regression among patients with an index FVC < 80% showed that, after controlling for potential confounders, those with significant decline had greater odds of an inpatient hospitalization compared to those with marginal decline/stable FVC (OR 2.851, 95% confidence interval [CI] 1.172, 6.936, p = 0.021) (Fig. 3; see Appendix Table A3 for full results). Among patients with an index FVC% ≥80%, those with significant decline had similar odds of an inpatient hospitalization compared to those with marginal decline/stable FVC (OR 1.109, 95% CI 0.472, 2.607, p = 0.812). Other measures that were significantly (p < 0.05) associated with an increased odds of an inpatient hospitalization included index year 2017 vs. 2016, use of baseline oxygen therapy, and presence of diagnosis codes for diseases of the urinary system and upper GI disorders (Table A3). Increased age and evidence of immunomodulator use were associated with significantly (p < 0.05) reduced odds of an inpatient hospitalization (Table A3).

In a sensitivity analysis, stepwise logistic regression (final list of covariates listed in Appendix Table A4) demonstrated that among patients with an index FVC% <80%, those with significant decline had greater odds of an inpatient hospitalization compared to patients with marginal decline/stable FVC (OR 2.659, 95% CI 1.139, 6.205, p = 0.024). Among patients with an index FVC% ≥80%, those with significant decline had similar odds of an inpatient hospitalization compared to those with marginal decline/stable FVC (OR 1.247, 95% CI 0.558, 2.782, p = 0.591).

## Discussion

We investigated the association between FVC% decline and HRU among patients with non-IPF chronic fibrosing ILD using EHR data. To our knowledge, our study is the first to examine the impact of FVC decline on likelihood of inpatient hospitalization in a real-world sample of patients with non-IPF chronic fibrosing ILD. We found that a relative decline in FVC of at least 10% over 6 months, was associated with significantly higher odds of inpatient hospitalization among patients starting at diminished FVC% values of < 80%. The association between decline in FVC and inpatient hospitalization was not statistically significant in patients with a baseline FVC value ≥ 80%. Our results underscore the importance of preventing further FVC decline in patients with non-IPF chronic fibrosing ILD, particularly in patients with already diminished FVC% values.

A previous study examined the relationship between declining lung function and HRU among patients with IPF. In that study, greater FVC decline was significantly associated with increased inpatient hospitalizations in the period after 6 months following IPF diagnosis [14]. In addition, decline in FVC was associated with risk of IPF progression, suspected acute exacerbations, and mortality. Our study provides additional evidence of the association between FVC decline and HRU, in a non-IPF chronic fibrosing ILD patient population. IPF is considered the prototypical chronic fibrosing ILD that is progressive [23], our study is a mix of progressive and non-progressive ILDs and FVC decline was still associated with inpatient hospitalization. Although the results were in the same direction for both studies, there are some notable differences in characteristics of the study population. Our study population was older (average age 64.7 years versus 61.1 years) and had a lower proportion of males (43.8% versus 68.4%). Most importantly, the baseline FVC% in our study was higher (80.2%), compared to a baseline FVC% of 60.4% in the Reichmann et al. study. However, it should be noted that the association between FVC decline and inpatient hospitalization was significant only among patients with baseline FVC < 80% in our study.

We found that 31% of the patients with non-IPF chronic fibrosing ILD had an inpatient hospitalization during a six-month follow-up period. This inpatient hospitalization rate was in line with another study that found the proportion to be 25% in IPF patients [24], and higher than in another study of IPF patients in which the rate was 15% [14]. However, the rate reported in the study by Reichmann et al. was for IPF-related inpatient hospitalizations and not for all-cause inpatient hospitalization as reported in our study. In addition to evaluating the relationship between inpatient hospitalization and FVC decline, studies within IPF and chronic fibrosing ILD have shown the relationship between FVC decline and other outcomes, including mortality and acute exacerbations [14, 25–30]. Although these outcomes were not evaluated in our study, these studies provide additional evidence of the importance of maintaining FVC on health outcomes in patients with chronic fibrosing ILDs.

Inpatient hospitalization contributes significantly to the economic burden of the healthcare system within the United States. A claims-based study examining the burden of progression in patients with non-IPF chronic fibrosing ILD showed that inpatient hospitalization costs contributed to approximately 47% of the total medical costs [31]. Other studies similarly have shown the significant contribution of inpatient hospitalizations to the overall health care costs of patients with IPF [16] and non-IPF chronic fibrosing ILD [3]. Although the focus of our study was on the impact of lung function decline on inpatient hospitalizations, future studies could evaluate the impact of decline in FVC on medical costs in patients with non-IPF chronic fibrosing ILDs.

The findings of this study should be considered within the limitations of the data and study design. Only patients with sufficient clinical activity were included, and thus patients who did not maintain care with the same health care organization or who did not have an encounter during the study period were not included in the sample. It is possible that patients who have sufficient encounters for inclusion in the study may receive some care from another health care system not captured in the clinical database. We used FVC% decline of at least 10% to define lung function decline based on criteria used in the INBUILD clinical trial. However, the INBUILD trial also used other measures besides FVC% decline to define progression, measures that were not available to us in our data source. Thus, the marginal decline/stable FVC cohort may have included patients who were progressing based on INBUILD criteria. Nevertheless, our results still showed a significant relationship between FVC decline and (all-cause) inpatient hospitalization in patients with diminished lung function further demonstrating the importance of preserving lung function. FVC measures during the post-index period were only available for a subset of patients, and the extent of missing spirometry information is not distinguishable from the lack of an administered test. Other measures of lung function that may be examined by health care providers to identify lung function decline, e.g., diffusing capacity for carbon monoxide, were not available for a sufficiently large sample of patients and were not included in this study. In addition, FVC measured at index and at approximately 6 months may not reflect the actual trajectory of the decline in between the observed measures as NLP algorithms may not perfectly capture all FVC values and attribute them to the correct date. Despite these limitations, EHR data continue to be a powerful data source. These data allow for examination of HCRU patterns and detailed clinical data in a “real world” setting, outside the highly controlled environment of clinical trials.

## Conclusions

The patients in this real-world retrospective study existed along the variable spectrum of functional decline due to chronic fibrosing ILD. Patients with significant decline in FVC value started with higher lung function on average than those with marginal decline/stable FVC value. For patients starting with FVC% <80%, a decline of at least 10% in FVC value over 6 months was associated with significantly increased odds of an inpatient hospitalization, a significant contributor to health care costs and burden among patients with non-IPF fibrosing ILD. These findings support the importance of preserving lung function among patients with fibrosing ILD.

### Electronic supplementary material

Below is the link to the electronic supplementary material.

Supplementary Material 1

## Data Availability

The information underlying the results presented in the study include administrative medical and pharmacy claims data, available from Optum, which cannot be broadly disclosed or made publicly available at this time. The disclosure of this data to third-party clients assumes existence of certain data security and privacy protocols, and execution of a standard license agreement that includes restrictive covenants governing the use of the data. Information about licensing data from Optum, see https://www.optum.com/content/dam/optum/resources/productSheets/Clinformatics_for_Data_Mart.pdf.

## Abbreviations

AHRQ: Agency for Healthcare Research and Quality
ANOVA: Analysis of variance
BIPI: Boehringer Ingelheim Pharmaceuticals
BMI: Body mass index
CCS: Clinical Classifications Software
CI: Confidence interval
COPD: Chronic obstructive pulmonary disease
ED: Emergency department
EHR: Electronic health record
ERA: Endothelin receptor antagonists
FVC: Forced vital capacity
HIPAA: Health Insurance Portability and Accountability Act
HRU: Healthcare resource utilization
ICD: International Classification of Diseases
ICMJE: International Committee of Medical Journal Editors
ICU: Intensive care unit
ILD: Interstitial lung disease
IP: Inpatient
IPF: Idiopathic pulmonary fibrosis
OCD: Optum Clinical Database
OR: Odds ratio
PDE: Phosphodiesterase
PH: Pulmonary hypertension
PPI: Proton pump inhibitors
SD: Standard deviation
sGC: Guanylate cyclase stimulators
US: United States
