# Supplementary material for: Impact of chronic fibrosing interstitial lung disease on healthcare use: association between fvc decline and inpatient hospitalization
Source: BMC Pulm Med. 2023 Sep 9;23:337. doi: 10.1186/s12890-023-02637-8 (PMC10492374; doi:10.1186/s12890-023-02637-8)
Supplement: Supplementary file 1 — Supplementary Material 1 [file 12890_2023_2637_MOESM1_ESM.docx]

**SUPPLEMENTARY INFORMATION (Appendix)**

**Table A1.** Codes for ILD

| **ICD-10 Code** | **Description** | **Requires Fibrosis Code*** |
| --- | --- | --- |
| D86.0 | Sarcoidosis of the lung | Y |
| D86.2 | Sarcoidosis of the lung with sarcoidosis of the lymph nodes | Y |
| J60 | Coal Workers' Pneumoconiosis | N |
| J61 | Pneumoconiosis due to asbestos and other mineral fibers | N |
| J62.0 | Pneumoconiosis due to other talc dust | N |
| J62.8 | Pneumoconiosis due to other dust containing silica | N |
| J63.0 | Aluminosis (of lung) | N |
| J63.1 | Bauxite fibrosis (of lung) | N |
| J63.2 | Berylliosis | N |
| J63.3 | Graphite fibrosis (of lung) | N |
| J63.4 | Siderosis | N |
| J63.5 | Stannosis | N |
| J63.6 | Pneumoconiosis due to other specified inorganic dusts | N |
| J64 | Unspecified pneumoconiosis | N |
| J66.0 | Byssinosis | Y |
| J66.1 | Flax-dressers' disease | Y |
| J66.2 | Cannabinosis | Y |
| J66.8 | Airway disease due to other specific organic dusts | Y |
| J67.0 | Farmer's lung | Y |
| J67.1 | Bagassosis | Y |
| J67.2 | Bird-fanciers' lung | Y |
| J67.3 | Suberosis | Y |
| J67.4 | Malt workers' lung | Y |
| J67.5 | Mushroom workers' lung | Y |
| J67.6 | Maple bark-strippers' lung | Y |
| J67.7 | Air conditioner and humidifier lung | Y |
| J67.8 | Hypersensitivity pneumonitis due to other organic dusts | Y |
| J67.9 | Hypersensitivity pneumonitis due to unspecified organic dust | Y |
| J68.0 | Bronchitis and pneumonitis due to chemicals, gases, fumes and vapors | Y |
| J68.1 | Pulmonary edema due to chemicals, gases, fumes and vapors | Y |
| J68.4 | Chronic respiratory conditions due to chemicals, gases, fumes and vapors | Y |
| J68.8 | Other respiratory conditions due to chemicals, gases, fumes and vapors | Y |
| J68.9 | Unspecified respiratory condition due to chemicals, gases, fumes and vapors | Y |
| J69.0 | Pneumonitis due to inhalation of food and vomit | Y |
| J69.1 | Pneumonitis due to inhalation of oils and essences | Y |
| J69.8 | Pneumonitis due to inhalation of other solids and liquids | Y |
| J70.1 | Chronic and other pulmonary manifestations due to radiation | Y |
| J70.3 | Chronic drug-induced interstitial lung disorders | Y |
| J70.4 | Drug-induced interstitial lung disorders, unspecified | Y |
| J70.8 | Respiratory conditions due to other specified external agents | Y |
| J70.9 | Respiratory conditions due to unspecified external agent | Y |
| J84.09 | Other alveolar and parieto-alveolar conditions | Fibrosis code |
| J84.10 | Pulmonary fibrosis, unspecified | Fibrosis code |
| J84.111 | Idiopathic interstitial pneumonia, not otherwise specified | Fibrosis code |
| J84.112 | Idiopathic pulmonary fibrosis | Fibrosis code |
| J84.113 | Idiopathic non-specific interstitial pneumonitis | Fibrosis code |
| J84.114 | Idiopathic non-specific interstitial pneumonitis | Fibrosis code |
| J84.116 | Cryptogenic organizing pneumonia | Y |
| J84.117 | Desquamative interstitial pneumonia | Fibrosis code |
| J84.17 | Other interstitial pulmonary diseases with fibrosis in diseases classified elsewhere | Fibrosis code |
| J84.2 | Lymphoid interstitial pneumonia | Fibrosis code |
| J84.89 | Other specified interstitial pulmonary diseases | Fibrosis code |
| J84.9 | Interstitial pulmonary disease, unspecified | Fibrosis code |
| M05.10 | RLD with RA of unspecified site | Y |
| M05.111 | RLD with RA in right shoulder | Y |
| M05.112 | RLD with RA in left shoulder | Y |
| M05.119 | RLD with RA in unspecified shoulder | Y |
| M05.121 | RLD with RA in right elbow | Y |
| M05.122 | RLD with RA in left elbow | Y |
| M05.129 | RLD with RA in unspecified elbow | Y |
| M05.131 | RLD with RA in right wrist | Y |
| M05.132 | RLD with RA in left wrist | Y |
| M05.139 | RLD with RA in unspecified wrist | Y |
| M05.141 | RLD with RA in right hand | Y |
| M05.142 | RLD with RA in left hand | Y |
| M05.149 | RLD with RA in unspecified hand | Y |
| M05.151 | RLD with RA in right hip | Y |
| M05.152 | RLD RA in left hip | Y |
| M05.159 | RLD with RA in unspecified hip | Y |
| M05.161 | RLD with RA in right knee | Y |
| M05.162 | RLD with RA in left knee | Y |
| M05.169 | RLD with RA in unspecified knee | Y |
| M05.171 | RLD with RA in right ankle and foot | Y |
| M05.172 | RLD with RA in left ankle and foot | Y |
| M05.179 | RLD with RA in unspecified ankle and foot | Y |
| M05.19 | RLD with RA of multiple sights | Y |
| M32.14 | Lung involvement in systemic lupus erythematosus | Y |
| M33.11 | Other dermatopolymositis, with respiratory involvement | Y |
| M33.21 | Polymyositis, with respiratory involvement | Y |
| M33.91 | Dermatomyositis, unspecified, with respiratory involvement | Y |
| M34.81 | Systemic sclerosis with lung involvement | N |
| M35.02 | Sicca syndrome with lung involvement | Y |

* To meet the requirement for two diagnosis codes, two "fibrosis codes" are sufficient. If code="Y", then a code listed as "fibrosis code" is
required for the second code. If code="N", then another code="N' or a "fibrosis code" is required for the second code.

**Table A2**. Procedure/Treatment Codes

| **Procedure** | **Code** | **Type** | **Description** |
| --- | --- | --- | --- |
| Lung Transplant | 33935 | CPT | Heart-lung transplant with recipient cardiectomy-pneumonectomy |
|  | 33933 | CPT | Backbench standard preparation of cadaver donor heart/lung allograft prior to transplantation, including dissection of allograft from surrounding soft tissues to prepare aorta, superior vena cava, inferior vena cava, and trachea for implantation |
|  | 32851 | CPT | Lung transplant, single; without cardiopulmonary bypass |
|  | 32852 | CPT | Lung transplant, single; with cardiopulmonary bypass |
|  | 32853 | CPT | Lung transplant, double (bilateral sequential or en bloc); w/o cardiopulmonary bypass |
|  | 32854 | CPT | Lung transplant, double (bilateral sequential or en bloc); with cardiopulmonary bypass |
|  | 32855 | CPT | Backbench standard preparation of cadaver donor lung allograft prior to transplantation, including dissection of allograft from surrounding soft tissues to prepare |
|  | 32856 | CPT | Backbench standard preparation of cadaver donor lung allograft prior to transplantation, including dissection of allograft from surrounding soft tissues to prepare |
|  | S2060 | HCPCS | Lobar lung transplantation |
|  | 0BYC0Z0 | ICD-10 Proc | Transplantation of Right Upper Lung Lobe, Allogeneic, Open Approach |
|  | 0BYC0Z1 | ICD-10 Proc | Transplantation of Right Upper Lung Lobe, Syngeneic, Open Approach |
|  | 0BYC0Z2 | ICD-10 Proc | Transplantation of Right Upper Lung Lobe, Zooplastic, Open Approach |
|  | 0BYD0Z0 | ICD-10 Proc | Transplantation of Right Middle Lung Lobe, Allogeneic, Open Approach |
|  | 0BYD0Z1 | ICD-10 Proc | Transplantation of Right Middle Lung Lobe, Syngeneic, Open Approach |
|  | 0BYD0Z2 | ICD-10 Proc | Transplantation of Right Middle Lung Lobe, Zooplastic, Open Approach |
|  | 0BYF0Z0 | ICD-10 Proc | Transplantation of Right Lower Lung Lobe, Allogeneic, Open Approach |
|  | 0BYF0Z1 | ICD-10 Proc | Transplantation of Right Lower Lung Lobe, Syngeneic, Open Approach |
|  | 0BYF0Z2 | ICD-10 Proc | Transplantation of Right Lower Lung Lobe, Zooplastic, Open Approach |
|  | 0BYG0Z0 | ICD-10 Proc | Transplantation of Left Upper Lung Lobe, Allogeneic, Open Approach |
|  | 0BYG0Z1 | ICD-10 Proc | Transplantation of Left Upper Lung Lobe, Syngeneic, Open Approach |
|  | 0BYG0Z2 | ICD-10 Proc | Transplantation of Left Upper Lung Lobe, Zooplastic, Open Approach |
|  | 0BYH0Z0 | ICD-10 Proc | Transplantation of Lung Lingula, Allogeneic, Open Approach |
|  | 0BYH0Z1 | ICD-10 Proc | Transplantation of Lung Lingula, Syngeneic, Open Approach |
|  | 0BYH0Z2 | ICD-10 Proc | Transplantation of Lung Lingula, Zooplastic, Open Approach |
|  | 0BYJ0Z0 | ICD-10 Proc | Transplantation of Left Lower Lung Lobe, Allogeneic, Open Approach |
|  | 0BYJ0Z1 | ICD-10 Proc | Transplantation of Left Lower Lung Lobe, Syngeneic, Open Approach |
|  | 0BYJ0Z2 | ICD-10 Proc | Transplantation of Left Lower Lung Lobe, Zooplastic, Open Approach |
|  | 0BYK0Z0 | ICD-10 Proc | Transplantation of Right Lung, Allogeneic, Open Approach |
|  | 0BYK0Z1 | ICD-10 Proc | Transplantation of Right Lung, Syngeneic, Open Approach |
|  | 0BYK0Z2 | ICD-10 Proc | Transplantation of Right Lung, Zooplastic, Open Approach |
|  | 0BYL0Z0 | ICD-10 Proc | Transplantation of Left Lung, Allogeneic, Open Approach |
|  | 0BYL0Z1 | ICD-10 Proc | Transplantation of Left Lung, Syngeneic, Open Approach |
|  | 0BYL0Z2 | ICD-10 Proc | Transplantation of Left Lung, Zooplastic, Open Approach |
|  | 0BYM0Z0 | ICD-10 Proc | Transplantation of Bilateral Lungs, Allogeneic, Open Approach |
|  | 0BYM0Z1 | ICD-10 Proc | Transplantation of Bilateral Lungs, Syngeneic, Open Approach |
|  | 0BYM0Z2 | ICD-10 Proc | Transplantation of Bilateral Lungs, Zooplastic, Open Approach |
|  | T8630 | ICD-10 Dx | Unspecified complication of heart-lung transplant |
|  | T8631 | ICD-10 Dx | Heart-lung transplant rejection |
|  | T8632 | ICD-10 Dx | Heart-lung transplant failure |
|  | T8633 | ICD-10 Dx | Heart-lung transplant infection |
|  | T8639 | ICD-10 Dx | Other complications of heart-lung transplant |
|  | T86810 | ICD-10 Dx | Lung transplant rejection |
|  | T86811 | ICD-10 Dx | Lung transplant failure |
|  | T86812 | ICD-10 Dx | Lung transplant infection |
|  | T86818 | ICD-10 Dx | Other complications of lung transplant |
|  | T86819 | ICD-10 Dx | Unspecified complication of lung transplant |
|  | Z4824 | ICD-10 Dx | Encounter for aftercare following lung transplant |
|  | Z48280 | ICD-10 Dx | Encounter for aftercare following heart-lung transplant |
|  | Z942 | ICD-10 Dx | Lung transplant status |
|  | Z943 | ICD-10 Dx | Heart and lungs transplant status |
| IPF | J84112 | ICD-10 Dx | Idiopathic pulmonary fibrosis |
| HRCT | 71250 | CPT | Computed tomography, thorax; without contrast material |
|  | 71270 | CPT | Computed tomography, thorax; without contrast material, followed by contrast material(s) and further sections |
| Pulmonary rehabilitation | S9473 | HCPCS | Pulmonary rehabilitation program, non-physician provider, per diem |
|  | G0424 | HCPCS | Pulmonary rehabilitation, including exercise (includes monitoring), one hour, per session, up to two sessions per day |
|  | 0948 | Revenue | Other Therapeutic Services - Pulmonary rehabilitation |
| Oxygen therapy | 31730 | CPT | Transtracheal (percutaneous) introduction of needle wire dilator/stent or indwelling tube for oxygen therapy |
|  | 4030F | CPT | Long-term oxygen therapy prescribed |
|  | E0424 | HCPCS | Stationary compressed gaseous oxygen system, rental; includes container, contents, regulator, flowmeter, humidifier, nebulizer, cannula or mask, and tubing |
|  | E0425 | HCPCS | Stationary compressed gas system, purchase; includes regulator, flowmeter, humidifier, nebulizer, cannula or mask, and tubing |
|  | E0430 | HCPCS | Portable gaseous oxygen system, purchase; includes regulator, flowmeter, humidifier, cannula or mask, and tubing |
|  | E0431 | HCPCS | Portable gaseous oxygen system, rental; includes portable container, regulator, flowmeter, humidifier, cannula or mask, and tubing |
|  | E0433 | HCPCS | Portable liquid oxygen system, rental; home liquefier used to fill portable liquid oxygen containers, includes portable containers, regulator, flowmeter, humidifier, cannula or mask and tubing, with or without supply reservoir and contents gauge |
|  | E0434 | HCPCS | Portable liquid oxygen system, rental; includes portable container, supply reservoir, humidifier, flowmeter, refill adaptor, contents gauge, cannula or mask, and tubing |
|  | E0435 | HCPCS | Portable liquid oxygen system, purchase; includes portable container, supply reservoir, flowmeter, humidifier, contents gauge, cannula or mask, tubing and refill adaptor |
|  | E0439 | HCPCS | Stationary liquid oxygen system, rental; includes container, contents, regulator, flowmeter, humidifier, nebulizer, cannula or mask, & tubing |
|  | E0440 | HCPCS | Stationary liquid oxygen system, purchase; includes use of reservoir, contents indicator, regulator, flowmeter, humidifier, nebulizer, cannula or mask, and tubing |
|  | E0441 | HCPCS | Stationary oxygen contents, gaseous, 1 month's supply = 1 unit |
|  | E0442 | HCPCS | Stationary oxygen contents, liquid, 1 month's supply = 1 unit |
|  | E0443 | HCPCS | Portable oxygen contents, gaseous, 1 month's supply = 1 unit |
|  | E0444 | HCPCS | Portable oxygen contents, liquid, 1 month's supply = 1 unit |
|  | E1390 | HCPCS | Oxygen concentrator, single delivery port, capable of delivering 85 percent or greater oxygen concentration at the prescribed flow rate |
|  | E1391 | HCPCS | Oxygen concentrator, dual delivery port, capable of delivering 85 percent or greater oxygen concentration at the prescribed flow rate, each |
|  | E1392 | HCPCS | Portable oxygen concentrator, rental |
|  | E1405 | HCPCS | Oxygen and water vapor enriching system with heated delivery |
|  | E1406 | HCPCS | Oxygen and water vapor enriching system without heated delivery |
|  | K0738 | HCPCS | Portable gaseous oxygen system, rental; home compressor used to fill portable oxygen cylinders; includes portable containers, regulator, flowmeter, humidifier, cannula or mask, and tubing |
|  | K0741 | HCPCS | Portable gaseous oxygen system, rental, includes portable container, regulator, flowmeter, humidifier, cannula or mask, and tubing, for cluster headaches |
|  | K0742 | HCPCS | Portable oxygen contents, gaseous, 1 month's supply = 1 unit, for cluster headaches, for initial month’s supply or to replace used contents |
|  | S8120 | HCPCS | Oxygen contents, gaseous, 1 unit equals 1 cubic foot |
|  | S8121 | HCPCS | Oxygen contents, liquid, 1 unit equals 1 pound |
|  | E0447 | ICD-10 Dx | Portable oxygen contents, liquid, 1 month's supply = 1 unit, prescribed amount at rest or nighttime exceeds 4 liters per minute (lpm) |
|  | Z9981 | ICD-10 Dx | Dependence on supplemental oxygen |
|  | 0277 | Revenue | Medical/Surgical Supplies and Devices - Take-home oxygen |
|  | 0413 | Revenue | Respiratory Services - Hyperbaric oxygen therapy |
|  | 0600 | Revenue | Home Health Oxygen - General |
|  | 0601 | Revenue | Home Health Oxygen - Stat/Equip/Supply or contents |
|  | 0602 | Revenue | Home Health Oxygen - Stat/Equip/Supply Under 1 LPM |
|  | 0603 | Revenue | Home Health Oxygen - Stat/Equip Over 4 LPM |
|  | 0604 | Revenue | Home Health Oxygen - Portable Add-on |
|  | 0609 | Revenue | Home Health Oxygen - Other |

**Table A3.** Full Logistic Model with Interaction for Any Inpatient Hospitalization between Index and 6 Months

|  | **Logistic Regression Model with Interaction (reference = marginal/stable and % predicted ≥ 80)** | | | | **Logistic Regression Model with Interaction (reference = marginal/stable and % predicted < 80)** | | | |
| --- | --- | --- | --- | --- | --- | --- | --- | --- |
|  | **Any IP Hospitalization between Index and 6 months** | | | | **Any IP Hospitalization between Index and 6 months** | | | |
|  | **odds ratio** | **lower 95% CI** | **upper 95% CI** | **p-value** | **odds ratio** | **lower 95% CI** | **upper 95% CI** | **p-value** |
| FVC decline and FVC percent predicted interaction |  |  |  |  |  |  |  |  |
| Marginal decline/stable and index FVC percent predicted < 80 | 1.660 | 1.030 | 2.677 | 0.037 | ref. | – | – | – |
| Significant decline and index FVC percent predicted < 80 | 4.733 | 1.856 | 12.069 | 0.001 | 2.851 | 1.172 | 6.936 | 0.021 |
| Marginal decline/stable and index FVC percent predicted ≥ 80 | ref. | – | – | – | 0.602 | 0.374 | 0.971 | 0.037 |
| Significant decline and index FVC percent predicted ≥ 80 | 1.109 | 0.472 | 2.607 | 0.812 | 0.668 | 0.293 | 1.525 | 0.338 |
| Age | 0.976 | 0.958 | 0.994 | 0.008 | 0.976 | 0.958 | 0.994 | 0.008 |
| Gender |  |  |  |  |  |  |  |  |
| Female | 0.865 | 0.559 | 1.339 | 0.515 | 0.865 | 0.559 | 1.339 | 0.515 |
| Male | ref. | – | – | – | ref. | – | – | – |
| Index year |  |  |  |  |  |  |  |  |
| 2016 | ref. | – | – | – | ref. | – | – | – |
| 2017 | 1.926 | 1.156 | 3.209 | 0.012 | 1.926 | 1.156 | 3.209 | 0.012 |
| 2018 | 1.605 | 0.922 | 2.793 | 0.094 | 1.605 | 0.922 | 2.793 | 0.094 |
| Race |  |  |  |  |  |  |  |  |
| Caucasian, non-Hispanic and Other/Unknown | ref. | – | – | – | ref. | – | – | – |
| Non-Caucasian/Caucasian, Hispanic | 0.660 | 0.340 | 1.282 | 0.220 | 0.660 | 0.340 | 1.282 | 0.220 |
| Region |  |  |  |  |  |  |  |  |
| Northeast and other/unknown | 0.970 | 0.590 | 1.597 | 0.906 | 0.970 | 0.590 | 1.597 | 0.906 |
| Midwest | ref. | – | – | – | ref. | – | – | – |
| South | 0.326 | 0.131 | 0.813 | 0.016 | 0.326 | 0.131 | 0.813 | 0.016 |
| West | 1.705 | 0.665 | 4.369 | 0.267 | 1.705 | 0.665 | 4.369 | 0.267 |
| Insurance Type |  |  |  |  |  |  |  |  |
| Commercial | 0.829 | 0.478 | 1.438 | 0.504 | 0.829 | 0.478 | 1.438 | 0.504 |
| Medicare/Medicaid | ref. | – | – | – | ref. | – | – | – |
| Commercial/Government | 1.403 | 0.817 | 2.407 | 0.219 | 1.403 | 0.817 | 2.407 | 0.219 |
| Other/Uninsured | 0.754 | 0.248 | 2.289 | 0.618 | 0.754 | 0.248 | 2.289 | 0.618 |
| Income |  |  |  |  |  |  |  |  |
| 0 - < $50,000 | ref. | – | – | – | ref. | – | – | – |
| $50,000+ | 0.466 | 0.242 | 0.896 | 0.022 | 0.466 | 0.242 | 0.896 | 0.022 |
| Index BMI |  |  |  |  |  |  |  |  |
| 0 - < 30 | ref. | – | – | – | ref. | – | – | – |
| 30+ | 1.037 | 0.594 | 1.809 | 0.899 | 1.037 | 0.594 | 1.809 | 0.899 |
| Missing | 1.286 | 0.719 | 2.300 | 0.397 | 1.286 | 0.719 | 2.300 | 0.397 |
| Diseases of the heart | 1.506 | 0.898 | 2.526 | 0.121 | 1.506 | 0.898 | 2.526 | 0.121 |
| Hypertension | 1.435 | 0.838 | 2.455 | 0.188 | 1.435 | 0.838 | 2.455 | 0.188 |
| Disorders of lipid metabolism | 0.582 | 0.326 | 1.040 | 0.068 | 0.582 | 0.326 | 1.040 | 0.068 |
| Diseases of the urinary system | 2.100 | 1.241 | 3.552 | 0.006 | 2.100 | 1.241 | 3.552 | 0.006 |
| Non-traumatic joint disorders | 0.834 | 0.466 | 1.494 | 0.542 | 0.834 | 0.466 | 1.494 | 0.542 |
| Diseases of arteries; arterioles; and capillaries | 1.516 | 0.860 | 2.673 | 0.150 | 1.516 | 0.860 | 2.673 | 0.150 |
| Diabetes mellitus without complication | 0.970 | 0.566 | 1.663 | 0.911 | 0.970 | 0.566 | 1.663 | 0.911 |
| Upper gastrointestinal disorders | 1.987 | 1.153 | 3.423 | 0.013 | 1.987 | 1.153 | 3.423 | 0.013 |
| Connective tissue disease | 0.861 | 0.450 | 1.647 | 0.651 | 0.861 | 0.450 | 1.647 | 0.651 |
| Asthma | 1.063 | 0.572 | 1.975 | 0.846 | 1.063 | 0.572 | 1.975 | 0.846 |
| Obstructive sleep apnea | 0.717 | 0.396 | 1.298 | 0.272 | 0.717 | 0.396 | 1.298 | 0.272 |
| Pulmonary hypertension | 0.600 | 0.280 | 1.287 | 0.190 | 0.600 | 0.280 | 1.287 | 0.190 |
| Heart failure | 0.814 | 0.391 | 1.698 | 0.584 | 0.814 | 0.391 | 1.698 | 0.584 |
| COPD | 1.580 | 0.982 | 2.543 | 0.059 | 1.580 | 0.982 | 2.543 | 0.059 |
| Lung cancer | 1.688 | 0.370 | 7.708 | 0.499 | 1.688 | 0.370 | 7.708 | 0.499 |
| Baseline oxygen therapy | 3.475 | 1.450 | 8.333 | 0.005 | 3.475 | 1.450 | 8.333 | 0.005 |
| Baseline high resolution CT (HRCT) | 0.908 | 0.539 | 1.531 | 0.718 | 0.908 | 0.539 | 1.531 | 0.718 |
| Immunomodulator | 0.400 | 0.173 | 0.925 | 0.032 | 0.400 | 0.173 | 0.925 | 0.032 |
| Corticosteroid | 1.019 | 0.625 | 1.661 | 0.940 | 1.019 | 0.625 | 1.661 | 0.940 |
| H2-antagonists and PPIs | 1.132 | 0.667 | 1.920 | 0.647 | 1.132 | 0.667 | 1.920 | 0.647 |
| Baseline all-cause count of ambulatory visits at 6 months | 0.945 | 0.894 | 1.000 | 0.049 | 0.945 | 0.894 | 1.000 | 0.049 |
| Baseline all-cause count of ER visits at 6 months | 5.869 | 0.658 | 52.364 | 0.113 | 5.869 | 0.658 | 52.364 | 0.113 |
| Baseline all-cause count of inpatient visits at 6 months | 6.741 | 0.706 | 64.391 | 0.097 | 6.741 | 0.706 | 64.391 | 0.097 |

*Notes*. Observations read = 566, Observations used= 566

Likelihood ratio: chi-square=127.182, DF=40, p-value=<0.001

Global Score: chi-square=118.175, DF=40, p-value=<0.001

Global Wald: chi-square=87.601, DF=40, p-value=<0.001

Hosmer and Lemeshow: chi-square=6.875, DF=8, p-value=0.550

Generalized R-Square = 0.201, max-rescaled R-Square = 0.283

Pseudo R-Square = 0.181

c statistic = 0.777

AIC-intercept: 703.684, AIC-intercept and covariates: 656.501

SC-intercept: 708.022, SC-intercept and covariates: 834.384

Specification link test: p-value=0.272

**Table A4.** Stepwise Logistic Regression Model of Any IP Hospitalization between Index and 6 Months

|  | **% Predicted Index FVC ≥ 80%** | | | | **% Predicted Index FVC < 80%** | | | |
| --- | --- | --- | --- | --- | --- | --- | --- | --- |
|  | **Any IP Hospitalization between Index and 6 months** | | | | **Any IP Hospitalization between Index and 6 months** | | | |
|  | **odds ratio** | **lower 95% CI** | **upper 95% CI** | **p-value** | **odds ratio** | **lower 95% CI** | **upper 95% CI** | **p-value** |
| FVC decline and FVC percent predicted interaction |  |  |  |  |  |  |  |  |
| Marginal decline/stable and index FVC percent predicted < 80 | 1.693 | 1.078 | 2.660 | 0.022 | – | – | – | – |
| Significant decline and index FVC percent predicted < 80 | 4.502 | 1.853 | 10.937 | <0.001 | 2.659 | 1.139 | 6.205 | 0.024 |
| Marginal decline/stable and index FVC percent predicted ≥ 80 | – | – | – | – | 0.591 | 0.376 | 0.928 | 0.022 |
| Significant decline and index FVC percent predicted ≥ 80 | 1.247 | 0.558 | 2.782 | 0.591 | 0.736 | 0.340 | 1.594 | 0.437 |
| Age | 0.981 | 0.967 | 0.996 | 0.014 | 0.981 | 0.967 | 0.996 | 0.014 |
| Index year |  |  |  |  |  |  |  |  |
| 2016 | – | – | – | – | – | – | – | – |
| 2017 | 2.101 | 1.287 | 3.428 | 0.003 | 2.101 | 1.287 | 3.428 | 0.003 |
| 2018 | 1.805 | 1.065 | 3.059 | 0.028 | 1.805 | 1.065 | 3.059 | 0.028 |
| Region |  |  |  |  |  |  |  |  |
| Northeast and other/unknown | 0.933 | 0.584 | 1.490 | 0.770 | 0.933 | 0.584 | 1.490 | 0.770 |
| Midwest | – | – | – | – | – | – | – | – |
| South | 0.378 | 0.159 | 0.899 | 0.028 | 0.378 | 0.159 | 0.899 | 0.028 |
| West | 1.910 | 0.796 | 4.583 | 0.147 | 1.910 | 0.796 | 4.583 | 0.147 |
| Income |  |  |  |  |  |  |  |  |
| 0 - < $50,000 | – | – | – | – | – | – | – | – |
| $50,000+ | 0.511 | 0.276 | 0.948 | 0.033 | 0.511 | 0.276 | 0.948 | 0.033 |
| Diseases of the urinary system | 1.978 | 1.215 | 3.218 | 0.006 | 1.978 | 1.215 | 3.218 | 0.006 |
| Upper gastrointestinal disorders | 1.889 | 1.180 | 3.024 | 0.008 | 1.889 | 1.180 | 3.024 | 0.008 |
| COPD | 1.651 | 1.074 | 2.538 | 0.022 | 1.651 | 1.074 | 2.538 | 0.022 |
| Baseline oxygen therapy | 2.273 | 1.080 | 4.782 | 0.030 | 2.273 | 1.080 | 4.782 | 0.030 |
| Immunomodulator | 0.382 | 0.176 | 0.829 | 0.015 | 0.382 | 0.176 | 0.829 | 0.015 |
| Baseline all-cause count of ER visits at 6 months | 5.649 | 0.788 | 40.485 | 0.085 | 5.649 | 0.788 | 40.485 | 0.085 |
| Baseline all-cause count of inpatient visits at 6 months | 3.963 | 0.606 | 25.915 | 0.151 | 3.963 | 0.606 | 25.915 | 0.151 |
| Observations read = 566, Observations used= 566 | | | |  |  |  |  |  |
| Likelihood ratio: chi-square=104.529, DF=17, p-value=<0.001 | | | | |  |  |  |  |
